# Pectus excavatum correction enhanced by pectoralis muscle transposition: A new approach

**DOI:** 10.1016/j.ijscr.2020.04.059

**Published:** 2020-05-11

**Authors:** Beatrice Aramini, Uliano Morandi, Giorgio De Santis, Alessio Baccarani

**Affiliations:** a Division of Thoracic Surgery, Department of Medical and Surgical Sciences, University of Modena and Reggio Emilia, Via Largo del Pozzo, 71, 41124, Modena, Italy; b Division of Plastic Surgery, University of Modena and Reggio Emilia, Largo Pozzo 71, 41124 Modena, Italy

**Keywords:** Pectus excavatum, Pectoralis muscle flap transposition, Modified Ravitch

## Abstract

Indications for the surgical correction of pectus excavatum include functional/physiological, cosmetic, and psychosocial reasons. The most popular open technique for pectus excavatum repair was proposed by Ravitch in 1949 as an open approach that requires partial resection of the costal cartilage, xiphoid excision, and osteotomy of the sternum. The goal is to remove abnormal rib cartilage while preserving the perichondrium, allowing regrowth of the rib cartilage to the sternum in a more anatomic manner. Operative technique. We present a case of bilateral pectoralis muscle flap transposition during a modified Ravitch procedure is presented herein. Conclusion: This approach allows for a significant reduction in late complications and improves both functional and aesthetic outcomes.

## Highlights

• Indications for the surgical correction of pectus excavatum.
• Pectus excavatum repair was proposed by Ravitch in 1949.
• The goal is to remove abnormal rib cartilage in a more anatomic manner.
• A case of bilateral pectoralis muscle flap transposition during modified Ravitch procedure.
• Significant reduction in late complications and better functional and aesthetic outcomes.

## 1 Introduction

**Fig. 1:**
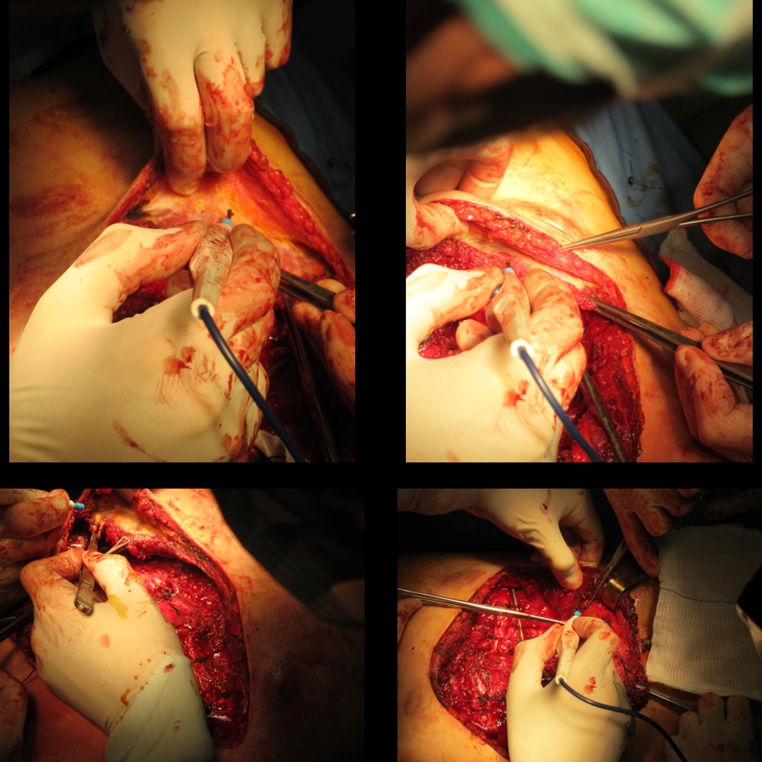
Muscle plane preparation after Ravitch procedure.

Pectus excavatum (PE) is the most common congenital chest wall deformity [1]. It is sometimes associated with cardio-respiratory impairment but is often related to psychological distress, particularly for patients in their teenage years [2]. One of the most successful techniques for open skeletal correction is the modified Ravitch [3]. Despite many surgical advances being described over time, major complications such as infection, wound dehiscence, and hardware exposure/dislocation, (which is sometimes visible under the skin, especially in patients with marfanoid habitus) may still compromise the final outcome [[4], [5], [6], [7], [8]]. Pectoralis muscle flap mobilization and transfer is a well-established reconstructive tool for plastic surgeons and represents a good solution for covering the osteotomized skeletal structures and hardware. A 24-year-old male underwent a modified Ravitch procedure for pectus excavatum with persistent tachycardia and dyspnea on exertion. Preoperative chest CT showed a severe pectus excavatum with a Haller index of 3.44. For the first time, the pectoralis muscle flap mobilization was associated with the modified Ravitch technique for pectus excavatum deformity correction (Fig. 1). This work has been reported in line with the SCARE criteria [9].

## 2 Operative technique

A clamshell incision is carried out approximately five centimeters below the nipple in males and at the inframammary fold in females. Dissection proceeds to the subcutaneous layer. The fascia is incised at the inferior border of the pectoralis muscles at the level of insertion of the rectus abdominis muscles. The pectoralis muscles are detached inferiorly from the ribs and sternum and are elevated with the skin and subcutaneous plane in one layer. The sternum and ribs are thus adequately exposed, taking advantage of the full length of the skin incision. The cartilage is removed from within the perichondrium by using electrocautery and thus resected with care taken to preserve the perichondrium. After the deformed cartilage is removed from the rib to the sternum, the xiphoid process is identified, resected, and elevated, and a blunt digital dissection of the posterior aspect of the sternum is achieved. The final sternum mobilization is obtained through a transversal osteotomy of its anterior cortical bone [5]. This sternal division is usually performed just above the beginning of the sternal depression. Occasionally, two sternal osteotomies are required to achieve adequate mobilization. This osteotomy is critical and must be performed carefully in a manner that preserves the continuity of the deep skeletal layer. A subxiphoid space is created, and the sternum is dissected from the underlying pericardium by electrocautery or blunt finger dissection. The intercostal bundles are then disconnected from the sternum and may be ligated or preserved. The sternum is elevated, and an anterior transverse wedge osteotomy is performed at the sternal-manubrial junction. The sternum is then osteotomized and elevated to a normal position. Sternal wires can aid in maintaining this position.

With the abnormal cartilage removed and the osteotomy performed, two appropriately sized pins are selected. The sternum is elevated anteriorly, and one pin is placed inside the sternum (Kirschner wire), with the other (Rush nail) being sutured or tied to the bilateral rib heads using absorbable sutures, such as PDS or Maxon.

**Fig. 2:**
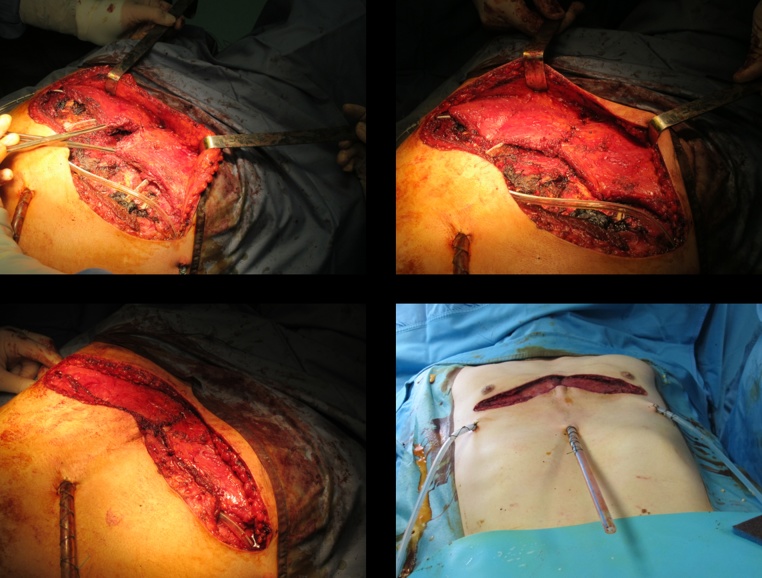
Pectoralis muscle transposition and medial suturing.

**Fig. 3:**
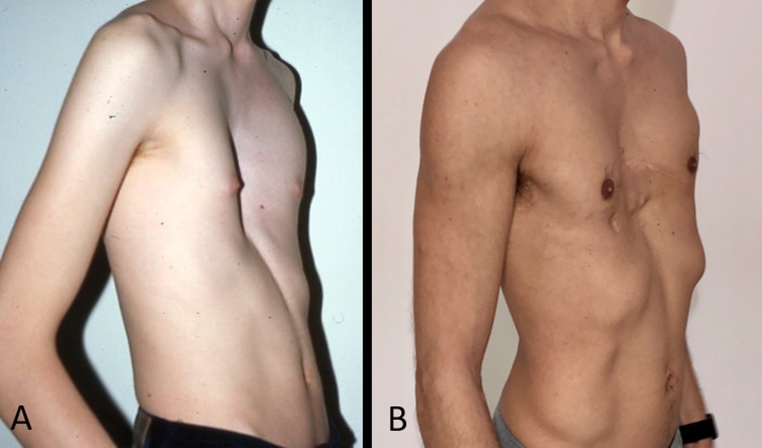
A. Appearance before modified Ravitch plus pectoralis muscle transposition. B. Results 6 months after surgery.

After the sterno-costal complex has been mobilized, elevated, and secured in an appropriate position with hardware, attention is paid to provide soft tissue coverage. Both pectoralis muscles are carefully dissected on a superficial prefascial plane from the overlying skin and subcutaneous layer. When proceeding cranially, care should be taken not to devascularize the skin flap. Skin bleeding and refilling is monitored accordingly while proceeding with the cranial dissection. Alternatively, skin perfusion may be intraoperatively assessed with the support of indocyanine Green angiography. Pectoralis muscles are elevated, and the thoraco-acromial pedicle is identified and preserved. Muscles are then mobilized as required to reach a comfortable lateral-to-medial rotation/transposition; to do so, both muscles are divided laterally from the humeral insertion, paying attention not to injure the thoraco-acromial pedicle. Once the flaps have been fully mobilized, hemostasis is accurately controlled, and the two flaps are sutured to one another medially with PDS sutures (Fig. 2). With this, full muscular coverage of the osteotomized sternum and ribs is obtained. Hardware is also almost fully protected by this maneuver. Two submuscular drains are inserted, and the muscles are sutured inferiorly to the deep fascia or to the rectus muscle fascia to obtain a complete muscular coverage of all the underlying elements. Final closure is thus obtained with skin sutures in a double layer. The results before and after surgery can be more effectively compared after some months (Fig. 3).

## 3 Conclusion

This approach combines for the first time the modified Ravitch procedure to a reconstructive surgery operation usually performed by plastic surgeons to close defects. According to our recently published series [6,8], this is a relatively straightforward procedure, and complications related to muscle transposition are limited. On the other hand, we consider that this approach significantly reduces mid-to-long term complications of the Ravitch procedure, such as hardware exposure, bone exposure, wound infection, and dehiscence, all of which may ultimately compromise the patient’s status [8].

No significant postoperative restrictions are necessary for the patient if not a limitation in pectoralis muscle contraction for the first month.

In our long-term experience with traditional open correction of pectus deformity with modified Ravitch, the few cases of wound infection were treated with multiple debridements associated with vacuum-assisted closure (VAC therapy) and final flap closure of the residual defect. This strategy has led us to devise our approach and transpose the muscle flaps since the first operation [8].

Despite the fact that the Nuss minimally invasive procedure is considered the standard in many centers, we believe that modified Ravitch should not be considered an "old fashion" technique, but just a technique, which needs to be addressed to the "appropriate" patient. Our experience is also supported by a recent a systematic review and meta-analysis published by Kanagaratnam A. et al. in 2016 [1]. In this study they considered Nuss procedure and Ravitch approach in 13 studies, comprising 1432 pediatric (79.3%) and adult (20.7%) patients, including 912 patients undergoing the Nuss procedure compared to 520 patients undergoing the Ravitch procedure, and they concluded that there was no significant differences between the Nuss group versus Ravitch group in pediatric patients with regard to overall complications, as wound infections, reoperations, hemothorax, pneumothorax or pneumonia. Additionally, adult patients undergoing the Nuss procedure had a higher incidence of overall complications. Interestingly, the final conclusion of this meta-analysis is that adult patients undergoing minimally invasive Nuss procedure had a higher incidence of overall complications, while in adults the Ravitch procedure resulted in fewer complications. Hence, the open approach and the pectoralis muscle transposition is recommended.

We are conscious of the “less reproducibility” of the Ravitch procedure in comparison to other techniques. However, we think that this approach requires adequate technical skills, specific experience and perhaps a quite long learning curve, but its use should be considered at least for severe deformities in adult patients.

In conclusion, we consider that this approach allows for a significant improvement of the final functional and aesthetic outcomes by providing a well-vascularized layer-protecting hardware, supporting bone and cartilage healing, and improving soft tissue thickness [6,8]. Furthermore, and less importantly, vascularized muscle represents an ideal site for adipose cell graft take in cases of need.

## Funding

Not applicable.

## Ethical approval

Ethical Board committee from the University Hospital of Modena (Italy) approved the study (242/2018/OSS/AOUMO).

## Consent

Written informed consent was obtained from the patient for publication of this case report and accompanying images. A copy of the written consent is available for review by the Editor-in-Chief of this journal on request.

## Author contribution

BA and AB wrote the manuscript; UM and GDS read and approved the manuscript.

## Registration of research studies

N/A.

## Guarantor

The authors are accountable for all aspects of the work in ensuring that questions related to the accuracy or integrity of any part of the work are appropriately investigated and resolved.

## Provenance and peer review

Not commissioned, externally peer-reviewed.

## Declaration of Competing Interest

The authors have no conflicts of interest to disclose.
